# The *Dunaliella salina *organelle genomes: large sequences, inflated with intronic and intergenic DNA

**DOI:** 10.1186/1471-2229-10-83

**Published:** 2010-05-07

**Authors:** David Roy Smith, Robert W Lee, John C Cushman, Jon K Magnuson, Duc Tran, Jürgen EW Polle

**Affiliations:** 1 Department of Biology, Dalhousie University, Halifax, NS, B3H 4J1, Canada; 2 Department of Biochemistry and Molecular Biology, MS200, 311B Fleischmann Agriculture, University of Nevada, Reno, NV 89557-0014, USA; 3 Chemical and Biological Process Development, Energy and Environment Directorate, Pacific Northwest National Laboratory, 902 Battelle Blvd, Richland, WA 99352, USA; 4 Department of Biology, Brooklyn College of the City University of New York, 2900 Bedford Ave, 200 NE, Brooklyn, NY 11210, USA

## Abstract

**Background:**

*Dunaliella salina *Teodoresco, a unicellular, halophilic green alga belonging to the Chlorophyceae, is among the most industrially important microalgae. This is because *D. salina *can produce massive amounts of β-carotene, which can be collected for commercial purposes, and because of its potential as a feedstock for biofuels production. Although the biochemistry and physiology of *D. salina *have been studied in great detail, virtually nothing is known about the genomes it carries, especially those within its mitochondrion and plastid. This study presents the complete mitochondrial and plastid genome sequences of *D. salina *and compares them with those of the model green algae *Chlamydomonas reinhardtii *and *Volvox carteri*.

**Results:**

The *D. salina *organelle genomes are large, circular-mapping molecules with ~60% noncoding DNA, placing them among the most inflated organelle DNAs sampled from the Chlorophyta. In fact, the *D. salina *plastid genome, at 269 kb, is the largest complete plastid DNA (ptDNA) sequence currently deposited in GenBank, and both the mitochondrial and plastid genomes have unprecedentedly high intron densities for organelle DNA: ~1.5 and ~0.4 introns per gene, respectively. Moreover, what appear to be the relics of genes, introns, and intronic open reading frames are found scattered throughout the intergenic ptDNA regions -- a trait without parallel in other characterized organelle genomes and one that gives insight into the mechanisms and modes of expansion of the *D. salina *ptDNA.

**Conclusions:**

These findings confirm the notion that chlamydomonadalean algae have some of the most extreme organelle genomes of all eukaryotes. They also suggest that the events giving rise to the expanded ptDNA architecture of *D. salina *and other Chlamydomonadales may have occurred early in the evolution of this lineage. Although interesting from a genome evolution standpoint, the *D. salina *organelle DNA sequences will aid in the development of a viable plastid transformation system for this model alga, and they will complement the forthcoming *D. salina *nuclear genome sequence, placing *D. salina *in a group of a select few photosynthetic eukaryotes for which complete genome sequences from all three genetic compartments are available.

## Background

*Dunaliella salina *Teodoresco [1] is one of the best-studied unicellular green algae [2-4]. This is not only because *D. salina *is halotolerant, thriving in extreme saline environments [3], but also because it can produce large quantities of β-carotene (up to 10% of the cell's dry weight) in lipid globules located within the chloroplast [5,6]. These traits make *D. salina *a model organism for investigating the evolution of salt adaptation [2] and an attractive "cell factory" for the commercial production of β-carotene [7,8]. Although a great deal is known about the physiology and biochemistry of *D. salina *[9,10], very little is known about the genomes it carries, especially those within its organelles. Until now, nothing was known about the size, conformation, or gene complement of either the mitochondrial or plastid genomes of *D. salina *(or those of any other *Dunaliella *species) even though the sequences of these genomes are essential to the development of new *D. salina *technologies, such as a viable plastid transformation system [11-16].

Research on green-algal organelle genomes has led to significant advancements in genetic engineering. The first stable transformation of a plastid genome was achieved in 1988 using the unicellular green alga *Chlamydomonas reinhardtii *[17] and, soon after, the first example of recombinant protein expression in a plastid was also achieved using *C. reinhardtii *[18]. Since then, many techniques for plastid engineering have been first developed for green algae and then adapted for use in land plants [19]. Given the relatively close evolutionary proximity of *C. reinhardtii *and *D. salina *[20], it is reasonable to assume that many of the technologies for *C. reinhardtii *plastid transformation might be transferable to *D. salina*. Various groups have attempted to transform *D. salina *[21]; however, a lack of plastid-genome sequence data has prevented successful plastid transformation. Therefore, the first step in developing an efficient and reliable plastid transformation system for *D. salina *is to sequence its organelle genomes.

*D. salina *is an attractive alga for organelle genome research and plastome engineering for a variety of reasons: i) various strains and geographical isolates of *D. salina *are readily available from algal culture collections around the world; ii) *D. salina *is relatively easy to grow and maintain -- it is one of the few microalgae that are being cultivated currently on a large scale; iii) *D. salina *lacks a rigid cell wall, facilitating organelle DNA extraction; iv) *D. salina *is unicellular, with only a single plastid, making it easier, as compared with multicellular species, to develop homoplasmic lines of plastid transformants; and v) being a close relative of the model green algae *C. reinhardtii *and *Volvox carteri*, means *D. salina *is an ideal species for comparative plant studies, especially comparative genomics, because the United States Department of Energy Joint Genome Institute (DOE JGI) is sequencing, or has sequenced, the *C. reinhardtii*, *V. carteri*, and *D. salina *nuclear genomes.

In 2006, the DOE JGI began sequencing the *D. salina *strain CCAP (Culture Collection of Algae and Protozoa) 19/18 nuclear genome, which is approximately 300 megabases (Mb) in length [[22]; DOE JGI, personal communication]. *D. salina *was selected for genome sequencing because of its potential as a feedstock producer for biofuels production [23] and its model status for studying saline adaptation. All of the *D. salina *whole genome shotgun sequencing (WGS) trace files that the DOE JGI produced are publicly available at the GenBank Trace Archive [24]; soon a complete assembly of the *D. salina *nuclear DNA (nucDNA) will be made public. The fact that *D. salina *is the third chlamydomonadalean alga for which there is a genome sequencing project, reaffirms that the Chlamydomonadales are emerging as the one of foremost lineages for comparative genomics.

The taxonomic position of the *Dunaliella *genus is still under debate [25]; however, it is often placed within the Chlamydomonadales (Chlorophyceae, Chlorophyta). For the purpose of this study, our definition of the Chlamydomonadales follows that of Lewis and McCourt [26], which includes the *Dunaliella *genus, and is equivalent to both the basal-bodies-clockwise group (CW group) [26] and the Volvocales *sensu *Nakada et al. [20]. Notably, some strains of *D. salina *were incorrectly identified in the past, which resulted in the deposition of inaccurately labeled DNA sequence data in public databases. Moreover, there exists a debate regarding the delineation of the species *D. salina *Teod. and *Dunaliella bardawil *Avron et Ben-Amotz [3,25]. According to Borowitzka and Borowitzka [27] and Borowitzka and Siva [28], the species *D. bardawil *is a *nomen nudum*; however, the name *D. bardawil *is still in use. Given the above issues, one should exercise great caution when using DNA sequence data in public databases that are said to have originated from *D. salina*.

As of November 1, 2009, complete and almost complete organelle DNA sequences are available for eight chlamydomonadalean algae, amounting to two plastid and eight mitochondrial genome sequences [29-38]; moreover, comprehensive genetic maps and limited sequence data are available for the plastid genomes of an additional four taxa [39-41]. The general features of these organelle genome data, as well as the species from which they are derived, are summarized in Table 1. Many of the available chlamydomonadalean organelle genome sequences are atypical in one way or another, having extreme sizes (e.g., large and expanded or highly compact [30,37]), unusual conformations (e.g., linear or linear fragmented [33,36,42]), and/or severely biased nucleotide composition (e.g., GC- or AT-rich [37,43]). Furthermore, there can be extensive size, conformational and/or compositional differences among the organelle genomes of closely related chlamydomonadalean species [36,41,43,44]. This wide assortment of genome architectures makes the Chlamydomonadales an ideal lineage for studying genome evolution [35,38,45,46].

**Table 1:** Available organelle-genome data for chlamydomonadalean algae.

Genus and species	Clade	Mapping conformation	Size (kb)	% **coding**^a^	%GC	GenBank Accession	Reference
**MITOCHONDRIAL GENOMES**							
*Chlamydomonas incerta*	*Reinhardtinia*	linear	~17.5^c^	~75^c^	~44^c^	DQ373068	[35]
*Chlamydomonas reinhardtii*	*Reinhardtinia*	linear	15.8-18.9^b^	67-82	~45	EU306617-EU306623	[29,30]
*Polytomella capuana*	*Reinhardtinia*	linear	13.0	82.0	57.2	EF645804	[36]
*Polytomella parva*	*Reinhardtinia*	linear-fragmented	16.2^d^	65.5^d^	41.0^d^	AY062933-AY062934	[33]
*Polytomella piriformis*^g^	*Reinhardtinia*	linear-fragmented	16.1^d^	65.8^d^	42.0^d^	GU108480-GU108481	*NA*
*Volvox carteri *f. *nagariensis*	*Reinhardtinia*	circular^f^	~35^c^	<40^c^	~34^c^	EU760701, GU084821	[37,38]
*Chlamydomonas eugametos*	*Moewusinia*	circular	22.9	53.4	34.6	AF008237	[31]
*Chlamydomonas moewusii*	*Moewusinia*	circular^e^	~21^e^	--	--	--	[39]
*Chlamydomonas pitschmannii*	*Moewusinia*	circular^e^	~16.5^e^	--	--	--	[40]
*Chlorogonium elongatum*	*Chlorogonia*	circular	22.7	53.3	37.8	Y13643-Y13644, Y07814	[32]
*Dunaliella salina*	*Dunaliellinia*	circular	28.3	42.0	34.4	GQ250045	this study

**PLASTID GENOMES**							
*Chlamydomonas gelatinosa*	*Reinhardtinia*	circular^e^	~285^e^	--	--	--	[41]
*Chlamydomonas reinhardtii*	*Reinhardtinia*	circular	204.2	43.3	34.5	FJ423446	[34]
*Volvox carteri *f. *nagariensis*	*Reinhardtinia*	circular	~525^c^	<20^c^	~43^c^	GU084820	[37,38]
*Chlamydomonas eugametos*	*Moewusinia*	circular^e^	~243^e^	--	--	--	[39]
*Chlamydomonas moewusii*	*Moewusinia*	circular^e^	~292^e^	--	--	--	[39]
*Chlamydomonas pitschmannii*	*Moewusinia*	circular^e^	~187^e^	--	--	--	[40]
*Dunaliella salina*	*Dunaliellinia*	circular	269.0	34.5	32.1	GQ250046	this study

Note: "--", data not available. Clades are defined by Nakada et al. [20].

^a ^Intronic open reading frames were not considered as coding DNA.

^b ^These data vary because of the presence/absence of optional introns.

^c ^These data are based on almost-complete genome sequences.

^d ^MtDNA consists of two fragments; data are based on the concatenation of these fragments.

^e ^These data are based on gel-electrophoresis and Southern-blot analyses.

^f ^The circular conformation of the *V. carteri *mtDNA is based on genome-assembly data [38] and needs to be confirmed by gel electrophoresis experiments.

^g ^This strain is formally known as *Polytomella *SAG 63-10.

Most of our knowledge of chlamydomonadalean organelle genomes comes from species within the *Reinhardtinia *clade (defined by Nakada et al. [20]). Indeed, six of the eight chlamydomonadalean algae for which significant organelle DNA sequence data are available come from this clade (Table 1), including *C. reinhardtii *and *V. carteri*. Given that the *Reinhardtinia *clade contains only a small fraction of the species diversity within the Chlamydomonadales, it would be intriguing to explore the organelle genomes of algae from other chlamydomonadalean clades. It would be particularly interesting to see if chlamydomonadalean algae from outside the *Reinhardtinia *clade have large, bloated plastid genomes. Both the *C. reinhardtii *and *V. carteri *plastid genomes, the only complete (or nearly complete) plastid DNA sequences that are available from the Chlamydomonadales, are among the largest and most noncoding-DNA dense plastid genomes observed to date, with sizes of 204 and ~525 kilobases (kb), respectively [34,38]. The forces driving these genomes towards distention are unknown, but they may be connected to the combined effects of a low mutation rate and a low effective population size [38].

Here we present the complete mitochondrial DNA (mtDNA) and plastid DNA (ptDNA) sequences of *D. salina *strain CCAP 19/18 -- a member of the *Dunaliellinia *clade [20]. The salient features of these genomes are described and compared with other chlamydomonadalean organelle genomes, particularly those of *C. reinhardtii *and *V. carteri*. The evolutionary and biotechnological implications of these sequences are discussed. The overreaching goals of this study are to use the *D. salina *organelle DNA data to test contemporary theories on genome evolution and to lay the foundation for a *D. salina *plastid transformation system.

## Results and discussion

### Overview of the *D. salina *organelle genomes

**Figure 1:**
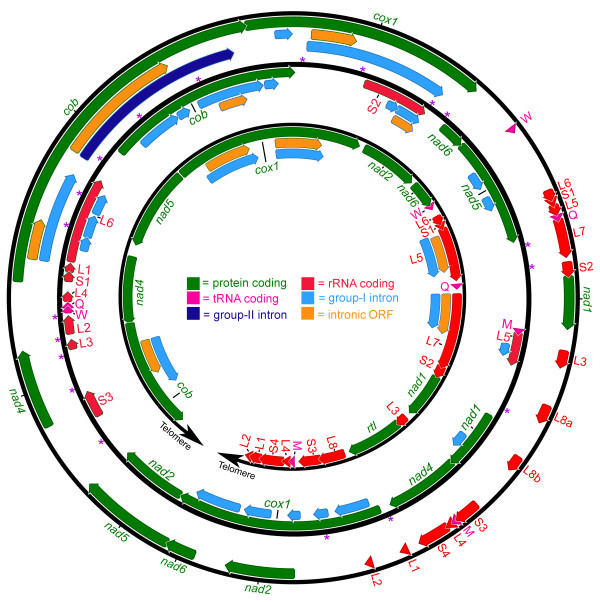
**Complete mitochondrial genome maps for *Dunaliella salina *(middle), *Chlamydomonas reinhardtii *(inner), and *Volvox carteri *(outer)**. The mitochondrial genome of *D. salina *(this study) is 28.3 kb, that of *C. reinhardtii *(GenBank accession numbers EU306617-EU306623) ranges from 15.8-18.9 kb, depending on the presence of optional introns, and that of *V. carteri *(GenBank accession numbers EU760701 and GU084821) is ~35 kb. Note that the *C. reinhardtii *mtDNA is a linear molecule. Arrows within the coding regions denote transcriptional polarities. The small subunit and large subunit rRNA-coding regions are fragmented into modules. Transfer RNA-coding regions are designated by the single-letter abbreviation of the amino acid they specify. Purple asterisks denote the sites of palindromic repeat clusters (see Figure 5 for more details). *Rtl *codes for a putative reverse-transcriptase-like protein.

The organelle genome sequences of *D. salina *were assembled using publicly available trace files that the DOE JGI *D. salina *nuclear genome sequencing project produced (see the Methods section for a detailed description of how the genome assembly was performed). Genetic maps of the *D. salina *organelle genomes are shown in Figures 1 (mtDNA) and 2 (ptDNA); for comparison, these two figures also include the corresponding genetic maps from *C. reinhardtii *and *Volvox carteri*. Table 1 outlines the general features of the *D. salina *organelle genomes, including their length, coding and noncoding DNA contents and nucleotide compositions, and compares these statistics to those from other chlamydomonadalean organelle DNAs. A Venn diagram highlighting the differences in gene content among available mtDNA sequences from the Chlamydomonadales is presented in Figure 3. A schematic compilation comparing the amounts of noncoding DNA in the *D. salina *organelle genomes with those from other completely sequenced (and almost complete) organelle genomes is shown in Figure 4, and analyses of the repetitive elements within the *D. salina *organelle DNA are summarized in Figure 5 and Supplementary Figures S1 and S2 [see Additional files 1 and 2].

**Figure 2:**
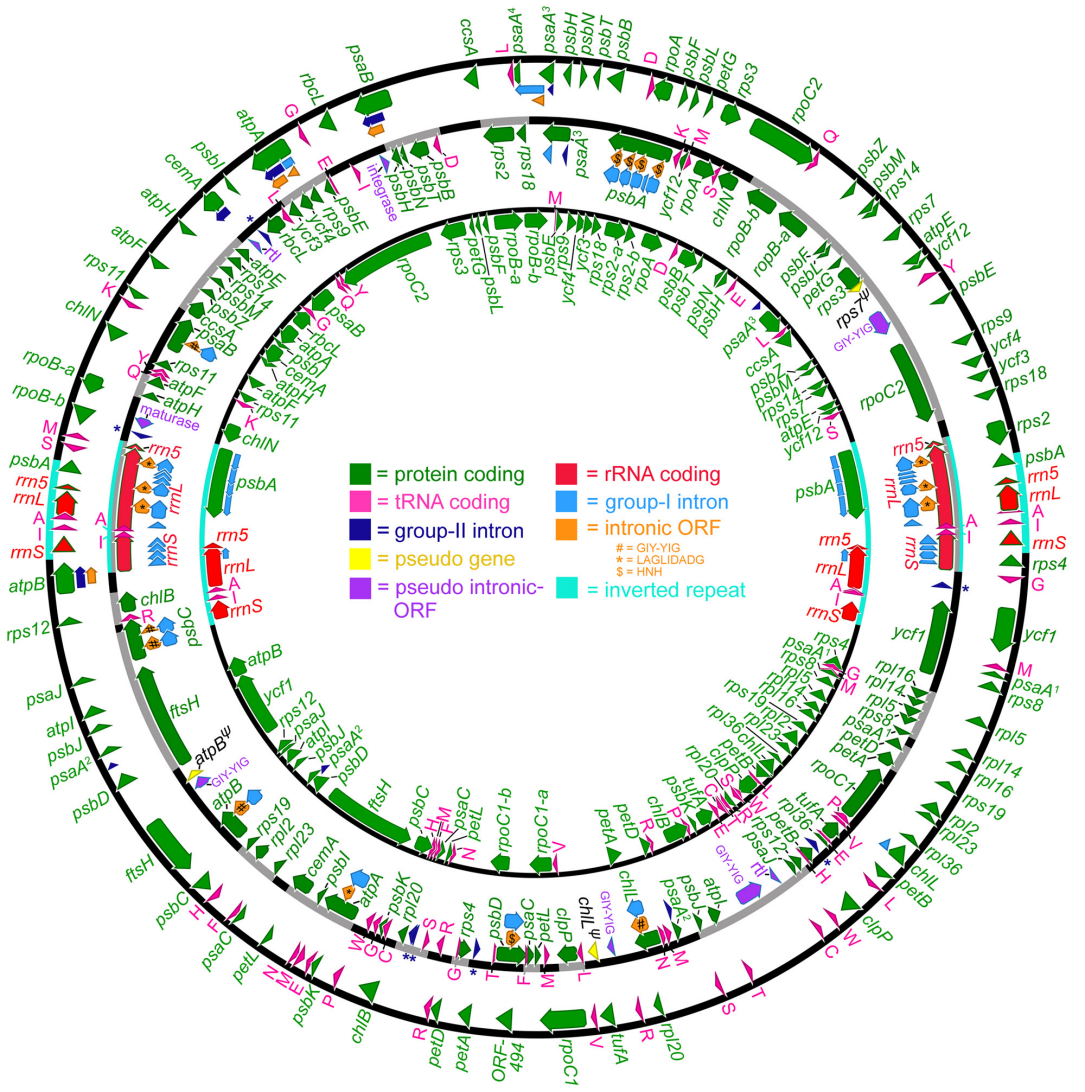
**Complete plastid genome maps for *Dunaliella salina *(middle), *Chlamydomonas reinhardtii *(inner), and *Volvox carteri *(outer)**. The *D. salina *plastid genome (this study) is 269 kb. The *C. reinhardtii *and *V. carteri *plastid genomes (GenBank accession numbers FJ423446 and GU084820) are 204.2 kb and ~525 kb, respectively. Arrows within the coding regions denote transcriptional polarities. Transfer RNA-coding regions are designated by the single-letter abbreviation of the amino acid they specify. Introns within intergenic regions are labeled with blue asterisks. Pseudogenes are labeled with a ψ. For all three genomes, the *psaA *gene is fragmented; the translational order of these fragments is set out using superscript numbers. The portions of the *D. salina *genome map that are gray (as opposed to black) highlight gene colinearity (not including introns) with either the *C. reinhardtii *or *V. carteri *plastid genomes.

**Figure 3:**
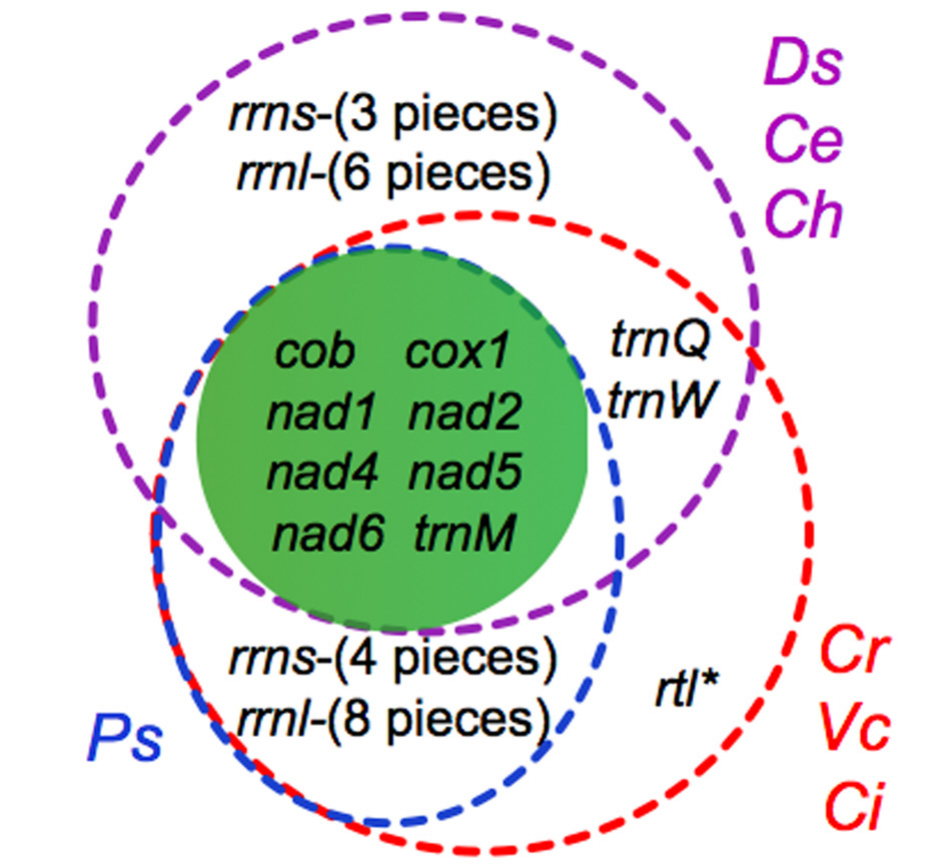
**Venn diagram comparing the gene repertoires of chlamydomonadalean mitochondrial genomes**. Chlamydomonadalean algae are labeled as follows: Ce = *Chlamydomonas eugametos*; Ch = *Chlorogonium elongatum*; Ci = *Chlamydomonas incerta*; Cr = *Chlamydomonas reinhardtii*; Ds = *Dunaliella salina*; Ps = *Polytomella capuana*, *Polytomella parva*, and *Polytomella piriformis *(strain SAG 63-10); Vc = *Volvox carteri*. *Rtl codes for a putative reverse-transcriptase-like protein: in *C. reinhardtii *and *C. incerta *this gene is independent of an intron, whereas in *V. carteri *it is within a group-II intron. Note, the *C. eugametos *mtDNA contains a duplicate copy of *trnM*.

**Figure 4:**
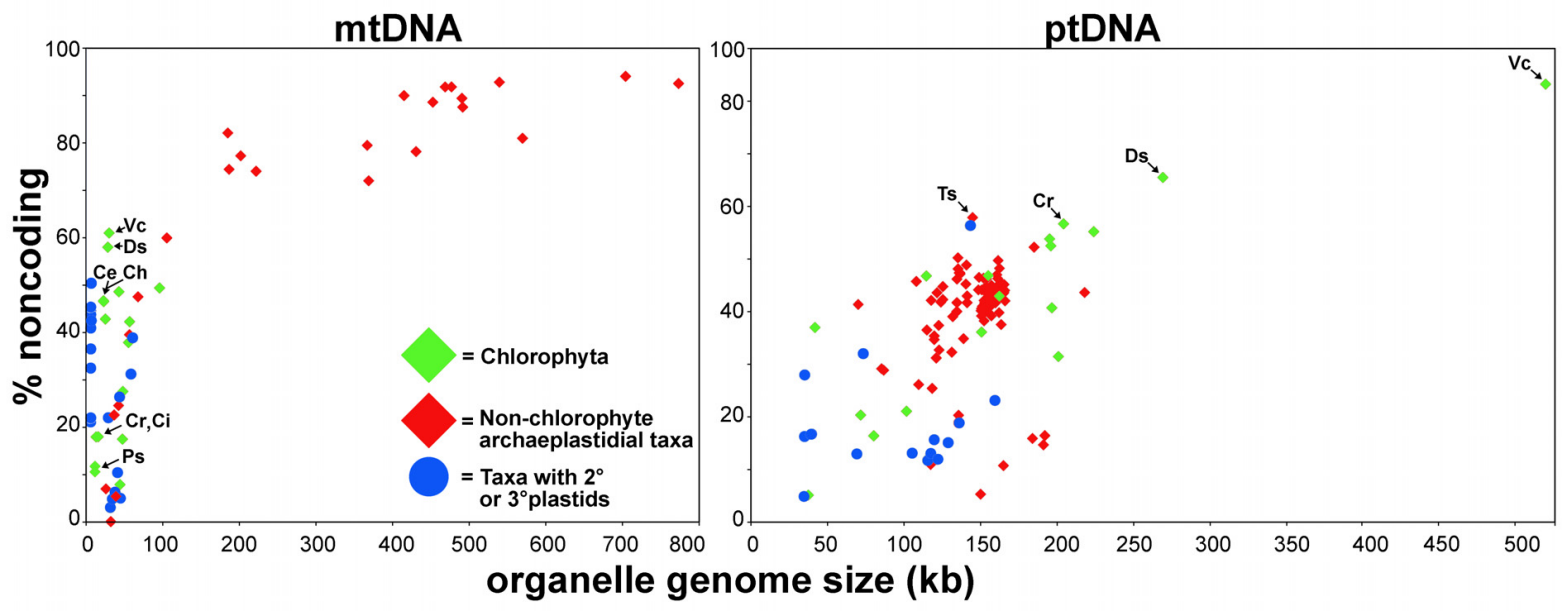
**Scaling of noncoding DNA content with genome size in completely sequenced organelle DNAs**. Chlamydomonadalean algae are labeled as follows: Ce = *Chlamydomonas eugametos*; Ch = *Chlorogonium elongatum*; Ci = *Chlamydomonas incerta*; Cr = *Chlamydomonas reinhardtii*; Ds = *Dunaliella salina*; Ps = *Polytomella capuana*, *Polytomella parva*, and *Polytomella piriformis *(strain SAG 63-10); Ts = *Trifolium subterraneum*; Vc = *Volvox carteri*.

**Figure 5:**
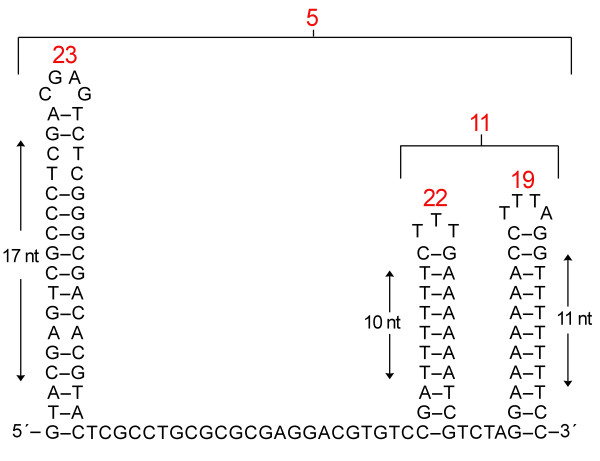
**Consensus sequences and secondary structures of the *D. salina *mitochondrial palindromic repeat elements**. The number of times each element appears in the *D. salina *mitochondrial genome is shown in red numbers. The locations of these palindromic elements within the mtDNA are depicted on Figure 1 using purple asterisks.

### Size, conformation, and nucleotide composition

The *D. salina *mitochondrial and plastid genomes are 28.3 and 269 kb, respectively, and assemble as circular molecules -- an observation that adds further support to the hypothesis that linear mitochondrial genomes in chlamydomonadalean algae are restricted to species within the *Reinhardtinia *clade (Table 1). The mitochondrial genome of *D. salina *is small relative to those of non-chlamydomonadalean green algae, which are, on average, 51.5 kb; however, it is still 5.4-15.3 kb larger than all other available chlamydomonadalean mtDNAs, except for that of *V. carteri*, which is ~35 kb ([37,38]). The size of the *D. salina *plastid genome is more pronounced than its mitochondrial counterpart, being the largest ptDNA sequenced thus far. Its nearest rivals are the 223.9 kb ptDNA of the chlorophycean green alga *Stigeoclonium helveticum *and the 217.9 kb plastid genome of the geranium *Pelargonium *× *hortorum *[47,48]. Large plastid genomes are a common theme among chlamydomonadalean algae: the *C. reinhardtii *plastid genome, at 204.2 kb [34], is the fourth largest completely sequenced ptDNA, partial sequence data indicate that the *V. carteri *ptDNA is ~525 kb in length ([37,38]), and gel electrophoresis results place the plastid genomes of *Chlamydomonas gelatinosa *(of the *Reinhardtinia *clade) and *Chlamydomonas moewusii *(of the *Moewusinia *clade [20]) at ~285 kb and ~292 kb in length, respectively [39,41]. The impressive size of the *D. salina *plastid genome (and those from other chlamydomonadalean algae) is a reflection of a prodigious noncoding DNA content rather than an unusually large gene repertoire. Within the *D. salina *ptDNA, a pair of inverted repeats, each with a length of 14.4 kb, divides the genome into a large (127.3 kb) and a small single-copy region (112.9 kb), referred to as the LSC and SSC regions. The *D. salina *inverted repeats are 6.2 kb shorter than their *C. reinhardtii *counterparts. This size discrepancy occurs because the *C. reinhardtii *inverted repeats contain *psbA*, a gene that is located in the SSC region of the *D. salina *ptDNA. Unlike in *D. salina*, the SSC and LSC regions of the *C. reinhardtii *ptDNA are virtually indistinguishable with sizes of ~80 kb. The precise lengths of the LSC, SSC, and inverted repeat regions for the *V. carteri *plastid genome are unknown; however, preliminary size estimates place them at >25 kb [38]. Southern blot analyses and partial sequence data indicate that the inverted repeats of the *C. moewusii *ptDNA may be upwards of 40 kb [39].

The GC content of the *D. salina *organelle DNAs is 34.4% (mtDNA) and 32.1% (ptDNA), which is unremarkable in relation to other archaeplastidial organelle genomes (i.e., those from eukaryotes with primary plastids). However, they are still the most GC-poor (or AT-rich) organelle DNAs observed within the Chlamydomonadales, which is significant because the Chlamydomonadales are one of the few lineages out of all eukaryotes known to contain species with GC-rich mitochondrial genomes (Table 1) [36,43]. The GC content among the different regions of the *D. salina *mitochondrial and plastid genomes is relatively constant: 33%_(*mtDNA*) _and 34%_(*ptDNA*) _for coding DNA; 34%_(*mtDNA*) _and 32%_(*ptDNA*) _for introns and intronic open reading frames (ORFs); and 37%_(*mtDNA*) _and 31%_(*ptDNA*) _for intergenic regions. For the different codon-site positions of the mtDNA and ptDNA protein-coding regions, the GC content is approximately 38%_(*mtDNA*) _and 42%_(*ptDNA*) _(1^st ^position); 38%_(*mtDNA*) _and 52%_(*ptDNA*) _(2^nd ^position); and 19%_(*mtDNA*) _and 13%_(*ptDNA*) _(3^rd ^position). Cumulative GC-skew analyses, (often used to pinpoint origins of replication [49]) of the *D. salina *mtDNA show a strong positive correlation with the transcriptional orientation (data not shown), reflecting the slightly higher GC content of the coding and intronic regions relative to the noncoding mtDNA. The same analysis of the ptDNA gives a more disordered plot, but one typical of ptDNA, because of the many shifts in transcriptional polarity throughout the genome.

### Coding content

Like other chlamydomonadalean species, *D. salina *has a severely diminished mtDNA gene content of only 12 genes, which represent seven proteins, two rRNAs, and three tRNAs. Outside of the Chlamydomonadales, the only species known to have more reduced mtDNA gene contents are found in the phyla Apicomplexa and Dinoflagellata and arguably some species within the supergroup Excavata [50,51]. Various studies have tried to explain why chlamydomonadalean algae have such reduced mtDNA gene contents [52,53], but at present no straightforward answer to this question exists. The *D. salina *mtDNA gene inventory mirrors those of *Chlorogonium elongatum *and *Chlamydomonas eugametos*, but it shows some differences to those of *Reinhardtinia-*clade algae. These differences, which can be visualized on the Venn diagram in Figure 3, involve changes in tRNA-coding content and in the number of rRNA-coding fragments found on the genome. For example, the *D. salina *mtDNA encodes three tRNAs, whereas *Polytomella *mtDNA contains only *trnM*, and the mitochondrial *rrns *and *rrnl *genes of *D. salina *are divided into three (S1-S3) and six (L1-L6) coding modules, whereas in available *Reinhardtinia-*clade mtDNAs the *rrns *and *rrnl *genes are fragmented into at least four and eight coding modules, respectively. Given the similarities among the *D. salina*, *C. elongatum*, and *C. eugametos *mitochondrial genomes, these findings add further appreciation for the stability of mtDNA gene content among chlamydomonadalean species outside of the *Reinhardtinia *clade and underscore the instability of mtDNA gene content among *Reinhardtinia-*clade taxa (Figure 3).

The *D. salina *plastid genome is much more gene rich than its mitochondrial counterpart, with 102 genes -- five of which are duplicates found in the inverted repeats. When ignoring these duplicates, there are a total of 66 protein-, 3 rRNA-, and 28 tRNA-coding genes. This gene content is reduced from those of green-plant species outside the Chlamydomonadales, which on average have 123 ptDNA-encoded genes, representing 85 proteins, 3 rRNAs, and 35 tRNAs. It appears that chlamydomonadalean algae, at some point during their evolution, went through a major reduction in ptDNA (and mtDNA) coding content relative to most other photosynthetic eukaryotes. The *D. salina *ptDNA gene repertoire is identical to those of *C. reinhardtii *and *V. carteri *with the following minor exceptions: i) the *D. salina *plastid genome encodes three copies of *trnI *-- one more than the *C. reinhardtii *and *V. carteri *ptDNAs; ii) *D. salina*, like *C. reinhardtii*, has two ptDNA copies of *trnE*, whereas *V. carteri *has only one; iii) for *D. salina *and *V. carteri*, the *rps2 *gene is represented by a single open reading frame, whereas for *C. reinhardtii*, *rps2 *is fragmented into two adjacent open reading frames (*rps2-a *and *rps2-b*); and iv) the *D. salina *ptDNA does not contain the *RoaA*-like gene (*orf494*), which is present in the *V. carteri *ptDNA. Preliminary investigations of the plastids from *Moewusinia-*clade algae [39,54] indicate that their ptDNA gene contents are similar to those of *D. salina*, *C. reinhardtii*, and *V. carteri*. Altogether, these findings suggest that ptDNA gene content is uniform throughout the Chlamydomonadales, save for some minor differences in the number of tRNA-coding genes.

### Gene order

All 12 genes on the *D. salina *mitochondrial genome are encoded on the same strand (i.e., have identical transcriptional polarities), a characteristic shared by the three other circular-mapping chlamydomonadalean mtDNAs sequenced thus far (Table 1). Sequence data from chlamydomonadalean algae whose mtDNAs map as linear molecules, such as *C. reinhardtii *and *Polytomella *spp., reveal genomes that have two unequally sized gene clusters (i.e., a group of two or more genes that are situated close to one another) with opposing transcriptional polarities, which proceed outwards toward the ends of the genome (Figure 1). Based on these and the above observations, it is reasonable to assume that the ancestral chlamydomonadalean mtDNA mapped as a circular molecule with ~12 genes, all of which were encoded on the same strand, and that the events giving rise to linear mtDNA were connected with, or resulted in, a shift in transcriptional orientation of approximately one-third of the genes. The mtDNA gene order of *D. salina *is unique and differs from those of other chlamydomonadalean algae. Very few conserved mtDNA gene clusters are shared among *D. salina *and other chlamydomonadalean species (Figure 1), but this is not surprising considering that mtDNA gene arrangements can vary significantly even among closely related species in this group [31,32,37,44]. Previous reports suggest that homologous or illegitimate recombination between mtDNA repeats is causing mitochondrial genome rearrangements in chlamydomonadalean algae [36,55,56]. The *D. salina *mitochondrial genome does contain minor amounts of repetitive DNA (discussed below); these repeats may be catalysts for genome rearrangements. In *C. reinhardtii*, mitochondrial genes are organized into operons, which are first transcribed into polycistronic primary transcripts and then subsequently processed into mature monocistronic units via endo- and exonucleolytic cleavage [29]. A scan of the *D. salina *mitochondrial genetic map reveals clusters of tightly packed genes separated by large stretches of noncoding DNA (Figure 1). These gene clusters may reflect the layout of operons in the genome, a theory supported by the fact that they are punctuated by regions of noncoding DNA that can be folded into secondary structures.

In contrast to the mtDNA, genes in the *D. salina *plastid genome are found on both strands and occur in small groups of two to four genes (Figure 2), which are distributed among the LSC and SSC regions and the inverted repeats (Figure 1). The former two regions contain approximately 50 and 45 genes, respectively, whereas the inverted repeats contain only five genes -- fewer than any chlamydomonadalean inverted repeat explored heretofore. The following additional genes are observed in the inverted repeats of other chlamydomonadalean algae: *psbA *(*C. reinhardtii*, *V. carteri, C. moewusii, C. eugametos, C. gelatinosa*, and *Chlamydomonas pitschmannii*), *rbcL *(*C. moewusii *and *C. eugametos*), and *atpB *(*C. gelatinosa*). Regions of gene synteny between the ptDNA of *D. salina *and those of *C. reinhardtii *and *V. carteri *(the only other chlamydomonadalean algae for which complete ptDNA maps are available) are highlighted in gray on Figure 2. The allocation of *D. salina *genes into small clusters is consistent with what is known for the *C. reinhardtii *plastid genome, where genes appear to be transcribed into monocistronic and dicistronic transcripts [57,58] rather than the larger polycistronic transcripts that are observed for the mtDNA. Thus, regions of gene colinearity between *D. salina *and *C. reinhardtii *(or *V. carteri*) may represent conserved transcriptional units.

### Introns and intergenic regions

One of the more salient features of the *D. salina *organelle genomes is their noncoding DNA content: 58% of the mtDNA and 65.5% of the ptDNA consist of either intergenic or intronic DNA. These values approach those of the *V. carteri *mitochondrial (>60% noncoding) and plastid (>80% noncoding) genomes, which are currently the most inflated organelle DNA sequences from the Chlorophyta (a phylum containing most of the identified classes of green algae [26]) (Figure 4). In fact, next to *V. carteri*, the *D. salina *ptDNA has a greater noncoding DNA composition than any other plastid genome sequenced to date, exceeding that of the legume *Trifolium subterraneum *(57.9%) and *C. reinhardtii *(56.7%) (Figure 4) [34,59]. However, one would expect the unsequenced plastid genomes of *C. gelatinosa *and *C. moewusii*, based on their estimated sizes [39,41], to have more noncoding DNA than *D. salina *but less than that of *V. carteri *(i.e., between 65-80% noncoding). Interestingly, both the mitochondrial and plastid genomes of *D. salina *have equally large noncoding DNA densities (58% vs. 65.5%). This observation goes against what is seen in *C. reinhardtii *where the mtDNA and ptDNA have opposing architectures (~20% vs. ~57% noncoding), but it is consistent with the *V. carteri *organelle genomes, which are both distended with noncoding DNA (>60%).

The noncoding DNA in the *D. salina *organelle genomes can be subdivided into two categories: intergenic regions, which make up 8.37 kb (29.5%) of the mtDNA and 139.65 kb (52%) of the ptDNA, and introns and intronic ORFs, which together represent 8.05 kb (28.5%) and 36.49 kb (13.5%) of mitochondrial and plastid genomes, respectively. For the *D. salina *ptDNA, it is sometimes difficult to distinguish between intergenic DNA and intronic DNA because intron-like sequences (including intronic ORFs) are found in many of the intergenic regions (Figure 2). Altogether, 18 putative group-I introns were found in the mtDNA (two of which contain intronic ORFs) and 43 putative introns were discerned in the ptDNA: 36 within genes (35 of group-I and 1 of group-II affiliation) and 7 within intergenic regions (all of group-II affiliation). See Figures 1 and 2 as well as Supplementary Table 1 [Additional file 3] for a comparison of the organelle genome intron content of *D. salina *with those of *C. reinhardtii *and *V. carteri*. Note, because of the inverted repeats, 11 of the 43 introns in the ptDNA are duplicates (the single-copy-intron count for the ptDNA is 32). Seventeen of the gene-located ptDNA introns contain ORFs (their families are shown on Figure 2), whereas no ORFs were found within the ptDNA intergenic introns. The remnants of eight intronic ORFs (pseudo ORFs) were found in the intergenic ptDNA regions; these pseudo ORFs, which are often located adjacent to intergenic introns (Figure 2), appear to be nonfunctional because they contain frameshifts in their coding regions. All eight of the pseudo ORFs show sequence similarity to genes that are typically found in either group-I or group-II introns (Figure 2), such as genes coding for integrase-, maturase-, reverse-transcriptase- and endonuclease-like proteins. Intergenic introns have been identified in other genomes [60,61], but until now they had never been observed in chlamydomonadalean organelle DNA, or, to the best of our knowledge, any other green-algal organelle genomes. Most of the intergenic introns are highly derived and could only be identified using domain V, which is the most conserved secondary structure element of group-II introns [62]. Further experiments will need to be performed to confirm that the intergenic introns are functional (i.e., removed from mature transcripts) rather than inert sequences. If they are functional, then it would imply that many of the intergenic regions of the *D. salina *plastid genome are transcribed. There is also the possibility that the individual intergenic introns represent the fragments of larger introns that assemble after translation (i.e., the RNA fragments come together via base-pairing to form larger RNA species that are capable of splicing). However, secondary structure modeling of the intergenic introns gave no obvious indications that this was the case.

The intron/gene ratios for the *D. salina *mitochondrial and plastid genomes are 1.5 and 0.42, respectively. These values are much larger than those of other chlamydomonadalean organelle genomes, which range from 0 to 0.75 for available mitochondrial genomes, and are less than 0.07 for the two available ptDNA sequences. Notably, the *D. salina *ptDNA intron/gene ratio exceeds the average value for land-plant mitochondrial genomes (~0.6) [63], which are considered to be among the most intron-dense organelle DNAs.

The *D. salina *organelle DNAs contain significantly more introns than their *C. reinhardtii *and *V. carteri *counterparts (Figures 1 and 2). In stark contrast to the 18 introns found in the *D. salina *mtDNA, *C. reinhardtii *and *V. carteri *have five (all group I) and four (two group I and one group II) mitochondrial introns, respectively. Moreover, for *C. reinhardtii *all five introns are "optional" and, as of yet, the maximum number found in a single strain is three [52]. A similar trend is observed for the ptDNA: when counting duplicate genes only once, the plastid genome of *C. reinhardtii *has six introns (five group I and one group II) and that of *V. carteri *has eight (three group I and five group II); both these values are significantly less than the 32 unique putative introns found in the *D. salina *plastid genome. Interestingly, for all of the genes that contain introns in the *C. reinhardtii *and *V. carteri *organelle DNAs, their homologues in *D. salina *also contain introns, with the exception of *cemA*, which is intronless in *D. salina *but contains a group-II intron in *V. carteri*. The number of introns per gene and the intron insertion sites can differ among these three algae.

Other notable features of the *D. salina *noncoding organelle DNA include three pseudogenes (*rps7^ψ^*, *atpB^ψ^*, and *chlL^ψ^*) in the plastid genome. These pseudogenes, whose functional copies are also present in the ptDNA, were classified as such, not because they contain frameshifts in their coding sequence or because they appear highly degenerate relative to their functional counterparts, but because they are missing the first half or first two-thirds of their coding sequences. Furthermore, *atpB^ψ ^*and *chlL^ψ ^*are located immediately downstream of their functional copies, and in both cases a group-I intron is sandwiched between the functional gene and the pseudogene (Figure 2).

### Repeats

Unlike the mtDNA, which is relatively devoid of repeats, the *D. salina *plastid genome abounds with repetitive elements. The difference in repeat content between the mitochondrial and plastid genomes can be visualized by comparing their respective dotplot similarity matrices, which are shown in Supplementary Figures S1 (mtDNA) and S2 (ptDNA) [see Additional files 1 and 2]. Looking at the ptDNA dotplot, it is apparent that the ptDNA repeats are found in intergenic regions, introns, and in some of the longer protein-coding genes, such as *ftsH*, *rpoC2*, and *ycf1*. Nucleotide BLAST analyses of the *D. salina *plastid genome indicate that there are upwards of 5,000 repeats in the ptDNA, forming approximately 100 repeat subclasses. With some exceptions, these repeats range from 30-60 nt in length and are 70-90% AT. Some shorter (10-20 nt) GC-rich repeat elements (>50% GC) were also identified. The high degree of sequence similarity among the different ptDNA repeats is attributable to homopolymer runs rather than a recurring sequence motif. The *C. reinhardtii *and *V. carteri *plastid genomes are also rich in repetitive DNA and Southern blot analyses suggest that the *C. gelatinosa *ptDNA is as well [34,37,41]. Presumably, *C. pitschmannii *plastid genome, the smallest ptDNA observed from the Chlorophyceae (~187 kb), contains fewer repeats than other chlamydomonadalean ptDNAs [40], implying a connection between repeat content and plastid genome size. In a general sense, the *D. salina *ptDNA repeats are analogous to the short dispersed repeats described for the *C. reinhardtii *ptDNA [34], but they lack the consistent motif of the palindromic elements of the *V. carteri *plastid genome [37].

The *D. salina *mtDNA dotplot reveals a mostly blank matrix, with the exception of some small diagonal lines, which correspond to palindromic repeats (i.e., repeats that can be folded into hairpin structures). Clusters of palindromic repeats are found in 18 different noncoding regions of the mitochondrial genome. The genomic breadth of these clusters can be seen in Figure 1, where purple asterisks pinpoint the precise location of these repeat clusters. A consensus sequence of one of the more frequently occurring clusters is depicted in Figure 5. The mean length of the palindromic repeat clusters is 110 nt and, on average, they have a GC content of 50%. Each cluster contains approximately three palindromic elements (i.e., three putative hairpin structures), and the individual palindromes within each cluster range from 23-38 nt in length, and can be either AT- or GC-rich (Figure 5). Ten of the eighteen intergenic regions that are found in the mtDNA contain either one or two palindromic clusters (Figure 1), an arrangement that suggests that the palindromes may play a role in gene processing. Palindromic repeats have been described in other chlamydomonadalean mitochondrial genomes, including those of *C. reinhardtii*, *V. carteri*, and *Polytomella capuana *[29,36,37,56], and in many cases chlamydomonadalean mtDNA palindromic repeats have been implicated in RNA processing [36,37,64]. A notable observation is that the mitochondrial palindromic repeats of both *D. salina *and *V. carteri *often contain the sequence 5'-TTTA-3' (or 5'-TTT-3') in the loops of their hairpin structures (Figure 5).

### Organelle DNA from other *D. salina *strains

Prior to this study, organelle DNA sequence data for *D. salina *was limited to 14 GenBank entries amounting to 6.7 kb of ptDNA, divided over six protein-coding loci (no mtDNA sequence data were available). Most of these 14 entries do not list the strains of *D. salina *that were used for sequencing and only a few are associated with published articles. Alignments of the CCAP 19/18 ptDNA data generated here with the *D. salina *ptDNA data available at GenBank reveal a significant amount of sequence divergence: pairwise nucleotide diversity values varied from 2.4% to 17.6%, which suggests that none of the 14 *D. salina *entries are derived from strain CCAP 19/18 and that at least some of the sequences at GenBank labeled *D. salina *are not the same species as CCAP 19/18. In comparison, the average silent-site ptDNA diversity among seven different North American isolates of *C. reinhardtii *was estimated to be 1.4% [46], and a recent study on seven *V. carteri *geographical isolates found π_silent _of the ptDNA to be ~0.065% [38]. The high degree of sequence diversity among *D. salina *GenBank entries appears to be a reflection of the known issue of misidentification of *Dunaliella salina *isolates. *Dunaliella *researchers should be mindful of the high levels of sequence divergence between CCAP 19/18 and the available *D. salina *GenBank entries if using these data for sequence-based studies, such as designing PCR primers.

### Paving the way towards plastid transformation

The development of a reliable, high-efficiency genetic transformation system for *D. salina *is an important objective for the *Dunaliella *research community, especially when considering the many industrial applications that this technology could provide. Genetic transformation of the *D. salina *nuclear genome has been successful [21,65]; however, a lack of ptDNA sequence data has prevented successful attempts at transforming the *D. salina *plastid genome -- although an unsuccessful attempt was made to transform the plastid of *Dunaliella tertiolecta *[12]. For *Dunaliella *species, there are significant advantages to plastid transformation over nuclear transformation (see Verma and Daniell [14] for a review), some of which relate to the fact that ptDNA is polyploid and experiences high levels of gene expression. Now that the *D. salina *ptDNA sequence is available, scientists will be able to use these data to develop plastid transformation vectors targeting specific regions of the *D. salina *ptDNA. Plastid transformation occurs via homologous recombination between an engineered vector and a selected region of the plastid genome. Moreover, promoter and 3' UTR regions of genes [13] can now be used to design vectors for *D. salina *with improved expression levels. In principle, transgenes can be integrated into any site of the plastid genome, but transcriptionally-active intergenic regions are ideal [14]. One of the most frequently used integration sites is the *trnI*-*trnA *intergenic spacer, which is found in the inverted repeat of most plastid genomes, including that of *D. salina*. Given its popularity, the *trnI-trnA *intergenic region would be an ideal site for early attempts at transforming the *D. salina *ptDNA. However, the discovery of intergenic introns in the *D. salina *ptDNA is an indication that many of the intergenic regions are transcriptionally active, which, if true, should allow for a diverse range of transformation targeting sites.

## Conclusion

The *D. salina *organelle genomes are large, intron-dense molecules comprised predominantly of noncoding nucleotides. Repetitive elements punctuate the noncoding regions of the mitochondrial and plastid genomes, but are much more prevalent in the ptDNA. Overall, the discovery of putative intergenic introns in the *D. salina *ptDNA adds a new layer of complexity to the diverse repertoire of organelle genome architectures found in the Chlamydomonadales. Already a model organism for synthesizing β-carotene and studying salt adaptation, *D. salina *is now an ideal species for investigating organelle genome expansion. The high level of repetitive elements found in the plastid genome of *D. salina *may mirror the high level of repetitive sequences that is expected to be present in the nucDNA (DOE JGI, unpublished data). Publication of the plastid genome of *D. salina *is expected to result in major advances of plastid engineering with generation and use of transgenic *D. salina *strains for a number of new applications in the fields of biofuels as well as in vaccine antigene and biopharmaceutical production. The complete sequence of the *D. salina *nuclear genome will be made available soon, placing *D. salina *in a group of a select few photosynthetic eukaryotes for which complete genome sequences from all three genetic compartments are available.

## Methods

### *D. salina *strain information

The organelle DNA sequence data presented in this study come from *D. salina *strain CCAP 19/18, which is maintained at the Culture Collection of Algae and Protozoa (CCAP) in Argyll, Scotland [66]. *D. salina *CCAP 19/18 originates from the hypersaline Hutt Lagoon in Western Australia.

### Assembly of the *D. salina *organelle-genome sequences

The complete mitochondrial and plastid genome sequences of *D. salina *were generated by collecting and assembling the publicly available mtDNA and ptDNA trace files that the DOE JGI *D. salina *nuclear genome sequencing project produced [22]. Trace files were data-mined from the National Center for Biotechnology Information (NCBI) *D. salina *Trace Archive [24] using the following complete organelle genome sequences as trace BLAST (blastn 2.2.21+) queries: the *C. elongatum *and *C. eugametos *mitochondrial genomes, and the *C. reinhardtii *and *V. carteri *mtDNAs and ptDNAs -- similar approaches to assembling organelle genomes have been used in other studies (e.g., Smith and Lee [46]; Voigt et al. [67]). The BLAST parameters were as follows: an expectation value (E-value) of 10; a word size of 11; match and mismatch scores of 2 and -3, respectively; and gap-cost values of 5 (existence) and 2 (extension). Trace files showing >80% sequence similarity to the BLAST queries were downloaded and then assembled with CodonCode Aligner Version 2.0.6 (CodonCode Corporation, Dedham, MA, USA), which employs the Phred, Cross-match, and Phrap algorithms for base calling, sequence comparison, and sequence assembly, respectively. Assemblies were performed with a minimum percent identity score of 98, a minimum overlap length of 500 nt, a match score of 1, a mismatch penalty of -2, a gap penalty of -2, and an additional first gap penalty of -3. Gaps in the assemblies were filled by trace file walking, which was carried out by using *D. salina *mtDNA and ptDNA trace files as BLAST queries against the *D. salina *trace archive -- a process that allows one to "walk" slowly in both directions along the contigs, thereby, filling in any gaps. The final assemblies of the *D. salina *mtDNA and ptDNA trace files gave complete mitochondrial and plastid genome sequences with greater than 50-fold coverage.

### Analyses of introns and repetitive DNA in the *D. salina *organelle genomes

Introns in the *D. salina *organelle genomes were detected, classified, and folded into secondary structures using RNAweasel [62] and Rfam [68]. Introns that were not detected by these programs were identified by their ability to be folded into suitable secondary structures. Intron/gene ratios were calculated by dividing the number of introns in the genome by the gene number; for the *D. salina *ptDNA, intergenic introns were included in this calculation. Dotplot similarity matrices were generated with JDotter (version 12.2.0) using a sliding window size of 50 [69]. Mfold [70] was employed for all secondary structure analyses.

The *D. salina *organelle-DNA sequences were initially scanned for repeats with REPuter [71] using the Hamming distance option and a minimal repeat size setting of 12 nt. Forward, reverse, complement, and reverse complement repeats were all considered under REPuter. More detailed analyses of the *D. salina *organelle genomes for repeats were performed by building a custom BLAST databank of the mtDNA and ptDNA sequences and then comparing (blastn version 2.2.21+) this databank with specific regions from the mitochondrial and plastid genomes using an E-value of 5, a word size of 7, a match score of 2, a mismatch penalty of -3, a gap open score of 5, and a extend value of 2.

### The fraction of noncoding DNA in completely sequenced organelle genomes

Completely sequenced organelle genomes were downloaded from the NCBI Reference Sequence (RefSeq) collection [72] on June 1, 2009. The coding and noncoding DNA contents of these sequences were calculated using the following methods and definitions: i) the number of coding nucleotides in the genome is equal to the collective length of all annotated protein-, rRNA-, and tRNA-coding regions -- not including the portions of these regions that are also annotated as introns; ii) the amount of noncoding DNA is the genome length minus the number of coding nucleotides; iii) the number of intergenic nucleotides is equal to the genome length minus the collective length of regions annotated as genes (including their introns and intronic ORFs); and iv) the amount of intronic DNA is equivalent to the number of noncoding nucleotides minus the number of intergenic nucleotides. The above methods and definitions are contingent on the authors of the GenBank records having properly annotated their entry. If coding regions or introns have been ignored or inaccurately annotated, coding and noncoding DNA content values will be incorrect. All records were quickly scanned for major errors, but due to the large number of organelle genomes deposited in GenBank, it was unfeasible to review all of the records thoroughly.

### GenBank accession numbers

The GenBank accession numbers of the *D. salina *organelle-genome sequences are GQ250045 (mtDNA) and GQ250046 (ptDNA).

## Authors' contributions

DRS analyzed the data and wrote the manuscript. RWL, JCC, JKM and JEP helped in interpreting the data and revising the manuscript. DT cultivated the *D. salina*, isolated the DNA, and helped annotate the genomes. JCC provided the nucleic acids used for organellar and genomic sequencing. JCC, JKM, and JEP are the external leaders of the DOE JGI *Dunaliella salina *CCAP 19/18 nuclear genome sequencing project. All authors have read and approved the final manuscript.

## Supplementary Material

Additional file 1 **Figure S1**. Dotplot similarity matrix of the *D. salina *mitochondrial genome. Click here for file

Additional file 2 **Figure S2**. Dotplot similarity matrix of the *D. salina *plastid genome. Click here for file

Additional file 3 **Table S1**. Intron content of the *D. salina*, *C. reinhardtii*, and *V. carteri *organelle genomes. Click here for file
