# Supplementary material for: The Dunaliella salina organelle genomes: large sequences, inflated with intronic and intergenic DNA
Source: BMC Plant Biol. 2010 May 7;10:83. doi: 10.1186/1471-2229-10-83 (PMC3017802; doi:10.1186/1471-2229-10-83)
Supplement: Additional file 1 — Figure S1. Dotplot similarity matrix of the D. salina mitochondrial genome. [file 1471-2229-10-83-S1.PDF]

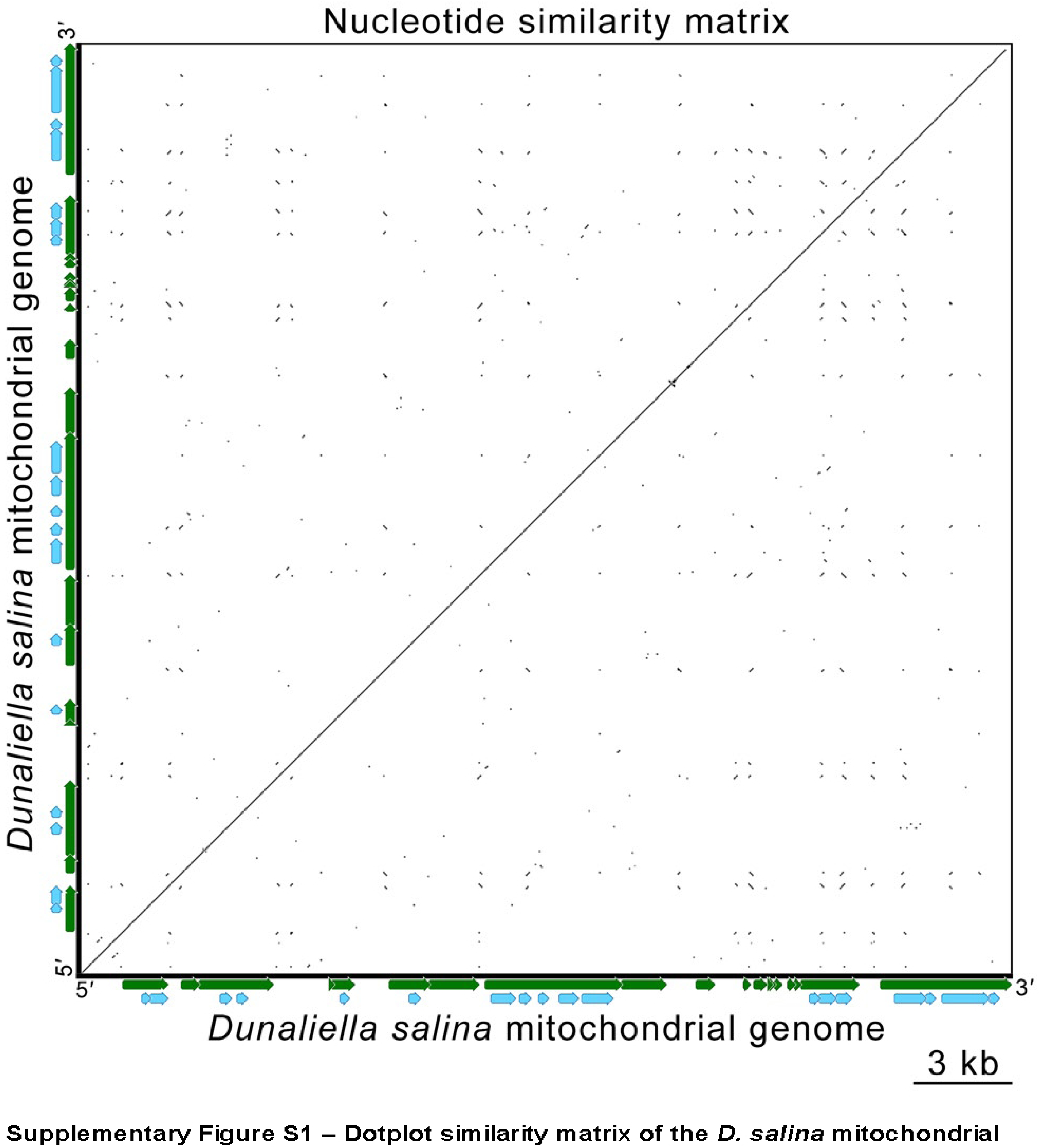

Supplementary Figure S1 – Dotplot similarity matrix of the *D. salina* mitochondrial genome.

The X- and Y-axes each represent the *D. salina* mitochondrial genome (28.3 kb). For clarity the genetic maps of the *D. salina* mtDNA are placed below and beside the axes — on these maps coding regions are green and introns are blue (refer to Figure 1 for the complete annotation). Dots in the nucleotide similarity matrix represent regions of sequence similarity. The matrix was generated using a sliding-window size of 50.
