# Supplementary material for: The Dunaliella salina organelle genomes: large sequences, inflated with intronic and intergenic DNA
Source: BMC Plant Biol. 2010 May 7;10:83. doi: 10.1186/1471-2229-10-83 (PMC3017802; doi:10.1186/1471-2229-10-83)
Supplement: Additional file 2 — Figure S2. Dotplot similarity matrix of the D. salina plastid genome. [file 1471-2229-10-83-S2.PDF]

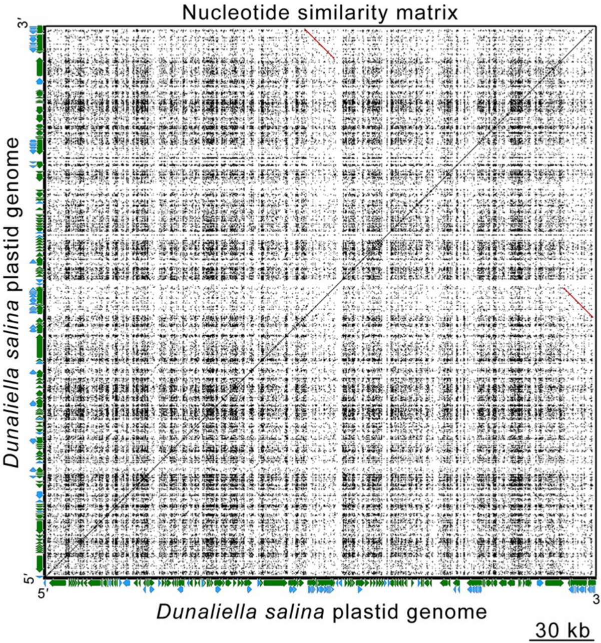

Supplementary Figure S2 – Dotplot similarity matrix of the *D. salina* plastid genome.
The X- and Y-axes each represent the *D. salina* plastid genome (260 kb). For clarity, genetic maps of the *D. salina* ptDNA are placed below and beside the axes — on these maps coding regions are green and introns are blue (refer to Figure 2 for the complete annotation). Dots in the nucleotide similarity matrix represent regions of sequence similarity. The matrix was generated using a sliding-window size of 50. The inverted repeats are highlighted in red in the matrix.
