# Supplementary material for: The Dunaliella salina organelle genomes: large sequences, inflated with intronic and intergenic DNA
Source: BMC Plant Biol. 2010 May 7;10:83. doi: 10.1186/1471-2229-10-83 (PMC3017802; doi:10.1186/1471-2229-10-83)
Supplement: Additional file 3 — Table S1. Intron content of the D. salina, C. reinhardtii, and V. carteri organelle genomes. [file 1471-2229-10-83-S3.PDF]

Supplementary Table S1 – Intron content of the *D. salina*, *C. reinhardtii*, and *V. carteri* organelle genomes. (Numbers of introns per gene are shown in brackets; insertion sites relative to the *C. reinhardtii* gene sequence are shown in red.)

Gene	*D. salina*		*C. reinhardtii*		*V. carteri*	
**MtDNA**	Group-I intron	Group-II intron	Group-I intron	Group-II intron	Group-I intron	Group-II intron
cob	(4) 429, 504, 756, 828	—	(1) 411[b]	—	(1) 411	(1) 822
cox1	(5) 189, 384, 711, 1110, 1281	—	(2) 384[b], 849[b]	—	(2) 700[b], 1089	—
nad1	(1) 600	—	—	—	—	—
nad2	—	—	—	—	—	—
nad4	—	—	—	—	—	—
nad5	(2) 663, 252	—	—	—	—	—
nad6	—	—	—	—	—	—
rrnL1	—	—	—	—	—	—
rrnL2	—	—	—	—	—	—
rrnL3	—	—	—	—	—	—
rrnL4	—	—	—	—	—	—
rrnL5	(1) 264	—	(1) 50[b]	—	—	—
rrnL6	(3) 259, 311, 398	—	—	—	—	—
rrnL7[a]	N/A	N/A	(1[b]) ~5[b]	—	—	—
rrnL8[a]	N/A	N/A	—	—	—	—
rrnS1	—	—	—	—	—	—
rrnS2	(2) 585, 622	—	—	—	—	—
rrnS3	—	—	—	—	—	—
rrnS4	—	—	—	—	—	—
**PtDNA**	Group-I intron	Group-II intron	Group-I intron	Group-II intron	Group-I intron	Group-II intron
atpA	(1) 492	—	—	—	(1) 492	(1) 756
atpB	(1) 1443	—	—	—	—	(1) 717
atpE	—	—	—	—	—	—
atpF	—	—	—	—	—	—
atpH	—	—	—	—	—	—
atpI	—	—	—	—	—	—
ccsA	—	—	—	—	—	—

cemA	—	—	—	—	(1)<br>351	—
chlB	—	—	—	—	—	—
chlL	(1)<br>876	—	—	—	(1)<br>201	—
chlN	—	—	—	—	—	—
clpP	—	—	—	—	—	—
ftsH	—	—	—	—	—	—
petA	—	—	—	—	—	—
petB	—	—	—	—	—	—
petD	—	—	—	—	—	—
petG	—	—	—	—	—	—
petL	—	—	—	—	—	—
psaA	(1)<br>2040	(2)<br>90, 270	—	(2)<br>90, 270	(1)<br>1605	(2)<br>90, 270
psaB	(1)<br>939	—	—	—	—	(1)<br>1920
psaC	—	—	—	—	—	—
psaJ	—	—	—	—	—	—
psbA	(5)<br>276, 384, 414, 570, 900	—	(4)<br>184, 204, 525, 726	—	—	—
psbB	—	—	—	—	—	—
psbC	(2)<br>543, 882	—	—	—	—	—
psbD	(1)<br>567	—	—	—	—	—
psbE	—	—	—	—	—	—
psbF	—	—	—	—	—	—
psbH	—	—	—	—	—	—
psbI	—	—	—	—	—	—
psbJ	—	—	—	—	—	—
psbK	—	—	—	—	—	—
psbL	—	—	—	—	—	—
psbM	—	—	—	—	—	—
psbN	—	—	—	—	—	—
psbT	—	—	—	—	—	—
psbZ	—	—	—	—	—	—
rbcL	—	—	—	—	—	—
rpl14	—	—	—	—	—	—
rpl16	—	—	—	—	—	—
rpl2	—	—	—	—	—	—
rpl5	—	—	—	—	—	—
rpl20	—	—	—	—	—	—
rpl23	—	—	—	—	—	—
rpl36	—	—	—	—	—	—
rpoA	—	—	—	—	—	—

rpoBa	—	—	—	—	—	—
rpoBb	—	—	—	—	—	—
rpoC1	—	—	—	—	—	—
rpoC2	—	—	—	—	—	—
rps2	—	—	—	—	—	—
rps3	—	—	—	—	—	—
rps4	—	—	—	—	—	—
rps7	—	—	—	—	—	—
rps8	—	—	—	—	—	—
rps9	—	—	—	—	—	—
rps11	—	—	—	—	—	—
rps12	—	—	—	—	—	—
rps14	—	—	—	—	—	—
rps18	—	—	—	—	—	—
rps19	—	—	—	—	—	—
rrn5	—	—	—	—	—	—
rrnL	(7) 276, 1902, 1994, 2322, 2509, 2561, 2659	—	(1) 2222	—	—	—
rrnS	(4) 415, 476, 740, 881	—	—	—	—	—
tufA	—	—	—	—	—	—
ycf1	—	—	—	—	—	—
ycf3	—	—	—	—	—	—
ycf4	—	—	—	—	—	—
ycf12	—	—	—	—	—	—
**PtDNA intergenic** [c]	Intron-like sequence		Intron-like sequence		Intron-like sequence	
rps3/rpoC2	(1)		—		—	
rrnS/ycf1	(1)		—		—	
rpl36/petB	(1)		—		—	
psaJ/atpI	(2)		—		—	
chlL/clpP	(1)		—		—	
psbD/rps4	(1)		—		—	
trnS/rpl20	(2)		—		—	
atpB/ftsH	(1)		—		—	
rrn5/atpH	(2)		—		—	
atpE/rbcL	(2)		—		—	
trnI/psbH	(1)		—		—	

Intron insertion sites are shown in red (*C. reinhardtii* was used as the reference genome for determining the nucleotide position of the intron insertion); dash (i.e., —) means no introns. As there are no introns in the organelle-DNA encoded tRNAs of *D. salina*, *C. reinhardtii*, and *V. carteri* only protein- and rRNA-coding genes are shown in the table.

[a] The LSU rRNA-coding regions of the *D. salina* mitochondrial genome are fragmented into six modules whereas those of *C. reinhardtii* and *V. carteri* are fragmented into eight coding modules (see Figures 1 and 3 for more details).
[b] Denotes optional intron.
[c] Refers to intron-like sequences found in the intergenic regions of the *D. salina* ptDNA (see Figure 2 for more details).
